# Factors Associated With Successful Enrollment in a Community Paramedicine Program for Heart Failure

**DOI:** 10.7759/cureus.34811

**Published:** 2023-02-09

**Authors:** Daniel Johnson, Jason Druschel, Brandon Wattai, Jessica Mann

**Affiliations:** 1 Department of Emergency Medicine, Penn State Health Milton S. Hershey Medical Center, Hershey, USA

**Keywords:** emergency medical services, transitional care, heart failure, mobile integrated healthcare, community paramedicine

## Abstract

Introduction

Participation in community paramedicine (CP) programs, sometimes referred to as Mobile Integrated Healthcare (MIH), may improve patient-centered outcomes and reduce hospital readmissions. The objective of this study was to correlate patient and system-specific factors with successful enrollment in a CP program for heart failure.

Methods

We conducted a retrospective review of patients enrolled in a CP program after hospitalization for a heart failure-related diagnosis. All patients greater than 18 years of age referred to the CP program with a heart-failure-related diagnosis were included. Factors including age, sex, hospital length of stay, enrollment method, concurrent use of transitional care services, care team, and service line referral were collected. The primary outcome was successful enrollment which led to an initial home visit. Chi-square and t-tests were performed to determine if the outcome differed between cohorts.

Results

A total of 908 patients met the inclusion criteria, and 677 (74.7%) received home visits. Increased participation was noted in patients enrolled in person (81.1% vs. 66%, p<0.01) and those also receiving transitional care services (78.9% vs. 62.5%, p<0.01).

Conclusion

We conclude that efforts should be made to contact patients in person, prior to hospital discharge, who are eligible for CP services.

## Introduction

Community paramedicine (CP), also commonly referred to as Mobile Integrated Healthcare (MIH), is a model of healthcare delivery that utilizes paramedics, outside of their traditional emergency response role, to provide healthcare resources to the community [1]. While each program is adapted to the individual community, they generally focus on underserved and high-risk patient populations [2]. The community paramedic, a common term for a CP practitioner, provides a wide range of services, from traditional medical assessment and care to health maintenance, education, and social services. Often, they connect patients, their healthcare team, and community-based resources to build a medical home for patients [3]. While CP addresses broad public health issues, many programs cater to a specific at-risk patient population. Common disease-specific examples include patients suffering from heart failure, chronic obstructive pulmonary disease, and diabetes. Studies have shown that significant portions of Medicare dollars are directed towards frequent hospitalizations related to complications of chronic diseases such as these [4]. For example, it is estimated that 23% of Medicare patients discharged from US hospitals between 2003 and 2006 after admission for a heart failure-related diagnosis were readmitted within 30 days [5]. Trends in the current literature support CP as a means to decrease hospital readmission and its associated morbidity and mortality [6]. Other common target populations include frequent utilizers of emergency medical services or the ED, with the notion that more valuable, efficient care can be provided elsewhere [7, 8]. The concept of adapting the traditional emergency medical services system to address the broad public health needs of the community was solidified in the 1996 EMS Agenda for the Future [9]. CP is a relatively novel concept that originated in the early 1990s, leaving much to learn about its benefits, pitfalls, and best practices. In 2012, a National Agenda for Community Paramedicine Research identified areas of particular research interest, primarily focusing on patient-centered outcomes. However, they also encouraged disseminating best practices to aid further program development [10]. Wide variation exists in the means of connecting patients to CP services. Some utilize large data sets to screen for patients who align with the program’s goals and objectives [11, 12]. Others leverage healthcare providers to recommend or refer their patients to a CP program [8, 13]. The method of contact and recruitment, whether in-person or remotely, also varies and is not well described in observational studies. Best practices are currently unknown. The objective of our study was to analyze program and patient-specific factors and their effect on successful enrollment in a CP program for heart failure.

## Materials and methods

Study design

This is a retrospective review of consecutive patients referred to a CP program for heart failure. Data regarding the patient’s demographics, preceding hospital stay, method of contact for enrollment, and care team were compiled. The primary outcome was successful enrollment in the program. For the purposes of this study, “successful enrollment” is used to describe the acceptance of CP services by the patient and the completion of an initial home visit. The study protocol was approved by Penn State University’s institutional review board (STUDY00009271).

Setting

The study was conducted at an academic, tertiary care referral center with an active heart failure cardiology service. The program aims to use paramedics with additional training to provide home visits for specific patient populations. The visits include disease-specific education, assistance with health system navigation, evaluation of the patient’s physical and social environment, and if needed, medical evaluation and treatment. Three full-time community paramedics perform these home visits, and an emergency medicine physician provides dedicated medical oversight. During the study period, the program only enrolled patients with specific heart failure-related diagnoses.

Inclusion and exclusion criteria

All adult patients (greater than or equal to 18 years of age) who were referred to CP and did not meet program exclusion criteria were included in the study. Patients who are discharged to post-acute rehabilitation facilities, long-term acute care facilities, skilled nursing facilities, or those who live greater than 90 minutes from the medical center are excluded from the program and are thus not included in the study.

Current practice

Patients are referred to the program in one of two ways. The electronic health record (EHR) is queried for patients with a heart failure-related diagnosis based upon a pre-determined list of ICD-10 codes (Table 1). Additionally, the discharging service can contact the CP team directly to request enrollment. Currently, attempts are made to approach patients prior to discharge from the hospital to introduce the program. This enrollment is completed by a community paramedic who also performs home visits. Patients who are discharged on weekends, after hours on weekdays, or when there is not a community paramedic available for enrollment are contacted by phone. If services are accepted, the community paramedic performs a home visit which includes a review of hospital discharge instructions, medication reconciliation, dietary counseling, weight monitoring, and general disease counseling. Protocols exist for IV diuresis and communication with the patient’s care team where necessary. There is no fee, and insurance is not billed for CP services. 

**Table 1:** International Classification of Disease (10th Revision) codes for included heart failure-related diagnoses.

ICD-10 codes for included heart failure-related diagnoses	
I11.0	Hypertensive disease with heart failure
I13.0	Hypertensive heart and chronic kidney disease with heart failure and stage 1 through stage 4 chronic kidney disease, or unspecified chronic kidney disease
I13.2	Hypertensive heart and chronic kidney disease with heart failure and with stage 5 chronic kidney disease, or end stage renal disease
I50.1	Left ventricular failure
I50.20	Unspecified systolic (congestive) heart failure
I50.21	Acute systolic (congestive) heart failure
I50.22	Chronic systolic (congestive) heart failure
I50.23	Acute on chronic systolic (congestive) heart failure
I50.30	Unspecified diastolic (congestive) heart failure
I50.31	Acute diastolic (congestive) heart failure
I50.33	Acute on chronic diastolic (congestive) heart failure
I50.41	Acute combined systolic (congestive) and diastolic (congestive) heart failure
I50.43	Acute on chronic combine systolic (congestive) and diastolic (congestive) heart failure
I50.9	Heart failure, unspecified

Data collection and analysis

Patient demographics and data regarding their home visits and preceding hospitalization were collected prospectively, in real-time, as part of an ongoing quality improvement and quality assurance process. These data were accessed retrospectively, after IRB approval, for the purposes of this study. Patient age, sex, enrollment method, concurrent use of transitional care services, care team, and service line referral were collected. A cohort of patients who were successfully enrolled was compared to a cohort who declined services. For continuous variables, a two-sample t-test was used to compare means. Chi-square analysis was used to compare categorical variables. A significant p-value is defined as less than 0.05. Analysis was performed using SAS (SAS Institute, Cary, NC).

## Results

From April 1, 2017 to March 31, 2018, a total of 908 patients with a heart failure-related diagnosis were contacted for participation in the CP program, with 679 completing initial home visits. Means of contact were not documented for two patients, and length of stay was not documented for one patient. Therefore, the total number of patients analyzed for these variables was 906 and 907, respectively. The median age was 70 years (IQR: 60-79). Forty-two percent were female. The most common admitting service was cardiology (434, 48%), followed by internal medicine (321, 35%). A total of 406 (45%) patients were contacted by telephone, with 268 (66%) accepting services resulting in an initial home visit. Of the 500 patients who had hospital contact prior to discharge, 409 (81.8%) completed an initial home visit resulting in an increased participation rate of 21.8% (p<0.01) compared to those contacted by phone. Patients also receiving transitional care services more frequently accepted CP services than those who did not (78.9% vs. 62.5%, p<0.01) (Table 2).

**Table 2:** Factors associated with decline or acceptance of initial home visit. a- Indicated patient was recruited, in person, prior to discharge from the hospital. b- Defined as a cardiologist within the health system. c- Hospital service that placed referral.

		Successful Home Visit		
	n	No	Yes	P-value
Age, Years, Median (IQR)	908	69 (60-79)	70 (60-80)	0.75
Length of Stay, Days, Median (IQR)	907	4 (3-7)	4 (3-8)	0.87
Discharge to Home Visit, Days, Median (IQR)	678		10 (5-23)	
Sex, n (%)	908			
Female		95 (24.9)	287 (75.1)	
Male		134 (25.5)	392 (74.5)	0.84
Inpatient Contact, n (%) ^a ^	906			
No		138 (34)	268 (66)	
Yes		91 (18.2)	409 (81.8)	<0.01
Transitional Care, n (%)	907			
No		86 (37.5)	143 (62.5)	
Yes		143 (21.1)	535 (78.9)	<0.01
In-network Cardiologist, n (%) ^b^	887			
No		31 (33.3)	62 (66.7)	
Yes		198 (24.9)	596 (75.1)	0.08
Service Line Referral, n (%) ^c^	906			
Cardiac Surgery		6 (35.3)	11 (64.7)	
Cardiology		97 (22.4)	337 (77.6)	
Emergency Medicine		0 (0)	9 (100)	
Family Medicine		21 (23.1)	70 (76.9)	
Hematology/Oncology		6 (37.5)	10 (62.5)	
Internal Medicine		95 (29.6)	226 (70.4)	
Neurology		1 (50)	1 (50)	
Neurosurgery		0 (0)	2 (100)	
Rehabilitation		1 (100)	0 (0)	
Pulmonary		1 (14.3)	6 (85.7)	
Vascular Surgery		1 (16.7)	5 (83.3)	

## Discussion

In this retrospective study, we sought to analyze factors related to successful enrollment in a CP program for heart failure. A significantly higher proportion of patients accepted services when they were approached by a community paramedic in person, prior to hospital discharge, versus after discharge via telephone (81.1% vs. 66%, p<0.01). There was also a higher likelihood of successful enrollment in patients who were concurrently enrolled in transitional care services (78.9% vs. 62.5%, p<0.01). Factors including age, sex, and hospital length of stay did not reach statistical significance. While the concept may seem obvious, patients can only realize the benefits of CP services if mechanisms are in place to enroll participants effectively. Little is currently known about the current practices of enrollment into CP programs at the system level. Much of the current data regarding enrollment practices are in the context of recruitment into trials rather than observational studies of the program itself. We could not identify any studies to date that directly compare enrollment methods with the outcome of subject participation. Nevertheless, acceptance of CP services in the literature varies widely. One trial was able to successfully enroll 77% of patients utilizing telephone contact [11], while others were only successful 37.6% of the time despite a process of in-person acquisition of informed consent [6]. Our data would suggest that in-person recruitment is associated with benefits. Similarly, upcoming randomized trials whose protocols have been published in advance plan to utilize in-person recruitment. One study seeks to utilize primary care physicians to advertise the program to improve enrollment [14], while another has staff dedicated to recruiting patients in EDs [7], both seeming to recognize the potential value of in-person recruitment.

CP is poised to be an integral part of interprofessional care for patients with complex healthcare needs. The research agenda and others prioritize investigating ideal ways to incorporate CP into the larger healthcare community [10, 15]. In a CP program for older adults, a core practice is the referral of their patients to healthcare resources [16]. In one program, 58.6% of participants were referred to at least one community-based health resource [17]. However, the relationship between CP and other community health resources should not be unidirectional. If such stakeholders are aware of the services that CP programs can provide, they can be instrumental in identifying and recruiting patients for enrollment. In our program, patients who concurrently were involved with the heart failure transitional care program in the health system were more likely to accept services, potentially due to this phenomenon. The success of CP programs may very well hinge upon the knowledge of their existence and referral from a broad range of health professionals [18]. While more patients with in-network cardiologists were successfully enrolled, this was not statistically significant. Cardiologists within the health system are likely more familiar with the CP resources locally and may endorse the program either based on word of mouth or positive experience. In a model program in Maine, they found that endorsement by a patient's primary care doctor increased enrollment into the CP program [13]. One plausible scenario is that a favorable view of CP by a patient's care team may be a driving factor for successful enrollment. Adequate dissemination of the presence and success of CP programs within the health system may be beneficial. Anecdotally, successes with our program's initial heart failure population led to the program's expansion and relationships with other service lines. CP programs have been associated with benefits related to patient and disease-specific outcomes, community health, and the well-being of the community paramedics themselves. Programs have demonstrated decreased ED visits, fewer inpatient stays, and decreased rates of 30-day readmission [17, 19-21]. Communities have observed decreased EMS utilization among high-utilizers [22], better access to care resources [17], and improved quality of life amongst its citizens [23]. The wellness of the community paramedics themselves also provides a unique perspective on the benefits of such programs. Traditional duties of paramedics in EMS systems are physically and mentally stressful. Transitioning to a role as a community paramedic can provide increased job satisfaction and professional development with the acquisition of new skills and knowledge and contribute to career longevity [24]. While the evidence grows supporting the benefit of such programs, the time has come to focus efforts on defining best practices in CP programs so that they can better serve their communities.

Limitations

Fundamentally, this was not a randomized controlled trial, and therefore potential exists for bias. Electronic queries lend themselves to errors in data entry. Incorrect reporting of diagnoses and methods of documenting primary and secondary diagnoses may cause patients to be missed. This sample certainly does not represent all patients who were admitted to the hospital with heart failure-related diagnoses during the study period that were eligible for CP services. Additionally, patients were referred for enrollment in a variety of ways. Endorsements by service lines that interact with CP more frequently may paint a more favorable overview of the program. Our data was not controlled for the source of referral. Patients who were discovered through the EHR and contacted by telephone potentially had no in-person discussion of the process by either a community paramedic or their in-patient care team. Finally, there is inherent variation in patients' investment in their healthcare needs, and participation in CP programs is voluntary. There are likely patient-specific factors that inherently contribute to their likelihood to accept services which were not accounted for in this study.

## Conclusions

Patients who were contacted in person prior to hospital discharge had a significant increase in the likelihood of successfully participating in the CP program. Additionally, it seems that those with relationships with other physicians or services within the health systems were successfully enrolled more frequently. We conclude that CP programs should build systems that include resources dedicated to in-person recruitment and enrollment. CP programs would also likely benefit from strong integration into the health system in order to leverage endorsements from other health care team members.
